# Targeting MDT Support: Which Patients Benefit Most in Outpatient Addiction Services?

**DOI:** 10.1192/bjo.2026.11536

**Published:** 2026-06-30

**Authors:** Mohammad Ali

**Affiliations:** Addictions Recovery Community (ARC-MK), Milton Keynes, United Kingdom

## Abstract

**Aims:**

Multidisciplinary team (MDT) input is widely recommended in outpatient addiction services to address complex needs, but its use and impact in routine practice are poorly understood. This study aimed to identify which patients receive MDT interventions and examine their outcomes compared with those who do not.

**Methods:**

A retrospective service evaluation was conducted in an outpatient addictions service over 12 months. All patients referred to the MDT for review were included (n=82), and compared with a matched cohort of patients not referred (n=130). Demographics, primary substance, comorbid mental health diagnoses, housing status, and treatment engagement were recorded. Functional outcomes (housing stability, employment/education, engagement with support services) were extracted from routine clinical records. Simple descriptive statistics and relative comparisons were used to identify patterns of MDT referral and benefit.

**Results:**

The MDT cohort had a mean age of 39 years; 64% were male. Primary substances included alcohol (41%), opiates (32%), stimulants (19%), and polysubstance use (8%). Comorbid mental health conditions were present in 57% of MDT patients versus 24% in non-MDT patients. Housing instability was more common in the MDT group (42% vs 18%). Functional improvements were observed in 61% of MDT patients, compared with 34% in non-MDT patients. The greatest gains were seen in patients with both comorbid mental illness and housing instability, particularly in engagement with social support services (46% vs 22% in non-MDT patients).

**Conclusion:**

MDT input in outpatient addiction services is selectively applied to patients with complex social and mental health needs. These patients demonstrate substantially higherfunctional gains than non-MDT patients, highlighting the value of targeted MDT interventions. Structured referral criteria and routine evaluation may help maximise MDT effectiveness and ensure equitable access.

